# Acute Liver Failure During Early Pregnancy—Case Report and Review of Literature

**DOI:** 10.3390/jcm14062028

**Published:** 2025-03-17

**Authors:** Banach Paulina, Justyna Kuczkowska, Yulia Areshchanka, Weronika Banach, Jakub Rzepka, Bartosz Kudliński, Rafał Rzepka

**Affiliations:** 1 Department of Gynecology and Obstetrics, Collegium Medicum, University of Zielona Góra, 65417 Zielona Góra, Poland; p.banach@cm.uz.zgora.pl (B.P.); j.kuczkowska@inm.uz.zgora.pl (J.K.); y.areshchanka@szpital.zgora.pl (Y.A.); 2 Poznan University of Medical Sciences, 61701 Poznań, Poland; weronika.wb98@gmail.com; 3 Department of Anesthesiology, Intensive Care and Emergency Medicine, Collegium Medicum, University of Zielona Góra, 65417 Zielona Góra, Poland; b.kudlinski@cm.uz.zgora.pl

**Keywords:** acute liver failure, pregnancy, intrahepatic cholestasis of pregnancy, primary biliary cholangitis, autoimmune hepatitis

## Abstract

**Background/Objectives:** This article presents the case of a 31-year-old primigravida who experienced acute liver failure in the 23rd week of pregnancy, along with a review of the literature on this rare condition during pregnancy. The purpose of this publication is to highlight the diagnostic and therapeutic challenges associated with acute liver failure in pregnant women. **Methods:** The patient presented with jaundice, pruritus, and dark-colored urine. Laboratory tests revealed a significant increase in aminotransferase, bilirubin, and bile acid levels, suggesting liver problems; however, due to the patient’s rapidly deteriorating condition and test results, autoimmune hepatitis was considered. Viral infections and other causes of liver damage were excluded. No clear diagnosis was established. The patient was administered ursodeoxycholic acid and due to her worsening condition, a cesarean section was performed at 23 weeks of gestation. After delivery, the patient’s condition improved, although she did experience cardiac arrest during hospitalization. The patient was discharged with a diagnosis of acute liver failure in the course of an overlap syndrome of autoimmune hepatitis and primary cholangitis or intrahepatic cholestasis of pregnancy. No abnormalities were noted during a follow-up visit 6 weeks after delivery. Despite a detailed case analysis, a final diagnosis was not established, which complicates planning for future pregnancies. **Discussion:** Several liver conditions can occur during pregnancy, including intrahepatic cholestasis of pregnancy, primary biliary cholangitis, and autoimmune hepatitis. Diagnosing these conditions can be challenging due to overlapping symptoms and metabolic and immunological adaptations during pregnancy that can affect the course of liver diseases. Rapid intervention is crucial to protect the health of both the mother and the fetus. **Conclusions:** In summary, this article aims to increase awareness of the complexities surrounding acute liver failure during pregnancy, highlighting the diagnostic challenges and importance of prompt medical intervention for the well-being of both the mother and the child. This paper aims to provide a comprehensive overview of the complexities surrounding acute liver failure during pregnancy, aiming to improve the understanding, diagnosis, and management of this condition.

## 1. Introduction

Acute liver failure (ALF) is a serious clinical condition that can arise from a variety of causes, including viral infections, toxins, medications, and autoimmune diseases. Patients with ALF may exhibit symptoms like jaundice, pruritus, dark urine, pale stools, nausea, vomiting, and signs of hepatic encephalopathy. These symptoms can manifest abruptly and worsen rapidly [1,2]. Depending on the etiology and severity, acute liver failure can lead to life-threatening conditions requiring urgent medical intervention, potentially including liver transplantation [3].

Pregnancy presents unique difficulties in diagnosing and treating acute liver conditions as this critical condition in pregnant patients requires urgent and comprehensive evaluation [1,2].

Pregnancy-related hormonal and metabolic changes can also unmask underlying liver disorders or trigger specific conditions, making the diagnosis of ALF challenging [1]. While intrahepatic cholestasis of pregnancy (ICP) is a more common liver disorder during pregnancy, characterized by pruritus and elevated bile acids, ALF presents a much more severe clinical picture with significant risks to both the mother and fetus [1,2,4].

ALF in pregnancy can stem from conditions unique to gestation, like acute fatty liver of pregnancy (AFLP) and HELLP syndrome, or it may be due to exacerbation of pre-existing conditions, such as autoimmune hepatitis or viral hepatitis [1,2]. It is also essential to consider drug-induced liver injury [1,2]. Treatment for ALF in pregnancy involves supportive intensive care, fetal monitoring, addressing the underlying cause, and in severe cases, may require early delivery or even liver transplantation [1]. It is important to distinguish ALF from other conditions, as pregnancy can unmask previously undiagnosed liver problems [5,6,7,8]. Autoimmune hepatitis (AIH) [6,7], primary biliary cholangitis (PBC) [1,4,5], and primary sclerosing cholangitis (PSC) [1,9,10] should be considered. These chronic cholestatic liver diseases may present during pregnancy, but their clinical course differs from ALF [1,5]. Also, atypical presentations of ICP in the first trimester should raise suspicion of other underlying causes [2]. Therefore, differential diagnosis in cases of acute liver disorders during pregnancy is crucial for optimal management and ensuring the safety of both the mother and child [1,5,8].

Acute liver failure during pregnancy is a medical emergency demanding a comprehensive diagnostic approach and rapid intervention. Due to potentially severe consequences for both the pregnant individual and the fetus, clinicians must be highly vigilant when encountering symptoms of liver dysfunction. Furthermore, pregnant women with pre-existing liver conditions should be closely monitored, as pregnancy may exacerbate their underlying disease [1,11]. Early diagnosis and appropriate treatment are crucial for improving maternal and fetal outcomes in cases of ALF during pregnancy [1,2,11].

This paper will discuss the case of a 31-year-old primigravida who experienced acute liver failure in the 23rd week of pregnancy, along with a review of the literature on the various aspects of acute liver failure, focusing on its causes, diagnosis, and therapeutic management, as well as its specific features in the context of pregnancy. Additionally, it will cover autoimmune liver diseases and their impact on the course of pregnancy.

## 2. Case Report

A 31-year-old female primigravida at 23 weeks and 2 days of gestation presented to the Obstetrics and Gynecology Department with significant yellowing of the skin, sclerae and mucous membranes of the body cavities, as well as a rash on the abdomen. She reported a history of darker-colored urine and mild pruritus for about two weeks, with no deterioration of her general condition. Previously, her pregnancy had been physiological, with no alarming symptoms, and no abnormalities were found in laboratory results during routine gynecological checks. Her family medical history was unremarkable.

On admission, the patient’s vital signs were normal; she was conscious and without signs of hepatic encephalopathy (HE). Her physical examination revealed yellowing of the skin and sclerae and a fine, macular rash on the abdomen. The abdomen was soft, non-tender, and Chelmonski’s sign as well as peritoneal signs were negative. General condition parameters were within the normal range. Imaging studies were performed. Ultrasonography confirmed fetal well-being, with a predicted fetal weight of 600 g. The ultrasound image of the abdomen was normal, and based on it, extrahepatic cholestasis was excluded.

The following laboratory tests were performed: morphology, PCT, CRP, potassium, sodium, iron, ferritin, creatinine, uric acid, TSH. Tests were performed to assess liver and pancreatic function (glucose, ALT, AST, total bilirubin, direct bilirubin, AFP, ceruloplasmin, amylase, albumin, cholesterol, lipase, fibrinogen, INR). Abnormalities of the following parameters were noted in the results of the above tests:

AFP 171.0 ng/mL (normal 0.89–8.78), ALT 1039.9 U/L (normal 10.0–31.0), AST 1162.1 U/L (normal 10.0–37.0), bile acids 219 umol/l (normal 2–10), direct bilirubin 12.95 mg/dL (normal 0.00–0.30), total bilirubin 15.21 mg/dL (normal 0.20–1.30), ferritin 347.00 ng/mL (normal 13.00–150.00), CRP 6.3 mg/L (normal 0.00–5.00). Based on negative results of amanitin, paracetamol and ammonia, as well as hepatitis A, B, CMV, EBV, and hepatitis E infections, poisoning and infectious diseases were excluded.

A preliminary diagnosis of “suspected gestational cholestasis” was made and treatment with ursodeoxycholic acid was implemented. On the second day of hospitalization, deteriorating liver parameters, INR of 1.83 (normal 0.85–1.25), prothrombin time of 1.83 s (normal 12–16), and PCT of 6.48 ng/mL (>2.0 high risk of sepsis and/or septic shock) were noted. Antibody results were obtained (ALD Profile-positive for Sp100, PML, and weakly positive for AMA). IgG, IgA, IgM, ANA, and Anti-LKM1 Ab turned out to be negative.

Due to the suspicion of acute autoimmune hepatitis, the patient was planned to qualify for liver transplantation.

Subsequent follow-up examinations revealed increasing liver failure (ALT of 1042 U/L, AST of 1214 U/L, bile acids of 283 umol/L, direct bilirubin of 15.05 mg/dL, total bilirubin of 17.71 mg/dL, and LDH of 248 U/l (normal 135.0–214.0)). The patient was qualified for delivery by emergency cesarean section (CS). At 23 + 3 weeks’ gestation, the patient had a CS under general anesthesia. A girl was born alive in poor general condition, APGAR 1-3-4-4, without acidosis (arterial cord blood pH 7.34).

After the cesarean section, the patient was transferred to the Intensive Care Unit, where she went into cardiac arrest 10 h after CS. Cardiopulmonary resuscitation was conducted for about 25 min. Advanced life support (ALS) procedures resulted in the return of spontaneous circulation (ROSC). The patient, in shock with vasoplegia, was disqualified from liver transplantation (did not meet the King’s College criteria).

On day 1 after cardiac arrest and after CS, follow-up imaging studies were performed: head CT scan (acute central nervous system pathologies were excluded), chest (no embolic features or emphysema after resuscitation), and abdominal cavity. Due to the described possible ischemia of the small intestine at a length of about 5 cm, the patient was qualified for an exploratory laparotomy. During the operation, the site of intestinal ischemia was not confirmed. In the following days of hospitalization, a substantial improvement in laboratory parameters was observed. Laboratory examinations conducted revealed signs of the patient’s improvement. ALT decreased from 556.3 U/L on day 1 to 199.4 U/L on day 15 after CS, while AST decreased from 571.4 U/L on day 1 to 170.6 U/L on day 15 after CS. Direct bilirubin levels dropped from 7.73 mg/dL on day 1 to 2.72 mg/dL on day 14 after CS, and total bilirubin decreased from 8.24 mg/dL on day 1 to 2.64 mg/dL on day 15 after CS. Additionally, LDH levels declined from 306 U/L after CS to 190.0 U/L on day 15.

After the 5th day of treatment in the Intensive Care Unit, the patient was transferred to the Internal Medicine Department with a gastroenterology subdivision for further diagnosis and treatment. The patient was discharged home on the 19th day of hospitalization with a diagnosis of acute liver failure in the course of an overlap syndrome of autoimmune hepatitis (AIH) and primary cholangitis (PBC) or intrahepatic cholestasis of pregnancy (ICP) with a recommendation for further diagnostics in the Hepatology Clinic.

The patient presented to the Obstetrics and Gynecology Clinic 6 weeks after delivery for a routine follow-up. No abnormalities were noted during the postpartum period or at the follow-up examination. Due to unremarkable laboratory results, as well as the absence of symptoms, the decision was made not to proceed with the biopsy and histopathological diagnosis. As a result, a definitive diagnosis was not established, nor was a management plan for subsequent pregnancies formulated.

The neonate was discharged from the Neonatology Ward on the 151st day of life with a weight of 3140 g and in good general condition.

### 2.1. Summary of Key Literature Findings

During the differential diagnosis of acute liver failure in pregnancy, autoimmune hepatitis (AIH), primary biliary cholangitis (PBC), and intrahepatic cholestasis of pregnancy (ICP) should be considered. Table 1 outlines the key distinguishing features of these conditions.

### 2.2. Indications for Cesarean Section

In this case, given the acute clinical course and deterioration of maternal health, the decision to proceed with an emergency CS was made. The indications for CS included the following:

1. predictive formulas indicating overlap syndrome and lack of liver biopsy;
2. the absence of liver biopsy results and the necessity for rapid intervention to preserve maternal and fetal health;
3. significant deterioration of maternal health and planned qualification for liver transplant, which would have not been possible during pregnancy.

## 3. Discussion

Pregnancy is a period when metabolism and immunological response undergoes intense adaptations [8]. This might cause the exacerbation of pre-existing entities, as well as new susceptibility to some diseases [8,12]. As the liver plays a major role in both maternal and fetal health, the bile acid disorders unmasked during gestation are of great importance [8].

A variety of conditions comprise cholestatic liver disease [13]. Cholestasis is a bodily disorder of the bile flow caused by either an obstruction in the biliary tract or a malfunction in bile acid formation or flow [13,14]. The bile canaliculi and intrahepatic bile ducts are involved in intrahepatic cholestasis, while extrahepatic cholestasis involves extrahepatic ducts, the common hepatic duct, or the common bile duct [13].

While considering a differential diagnosis of pregnant patients presenting with pruritus and jaundice, particular focus should be put on few specific entities. These include autoimmune hepatitis, primary biliary cholangitis, and intrahepatic cholestasis of pregnancy.

### 3.1. Autoimmune Hepatitis (AIH)

AIH is a rare immune-mediated chronic liver disease that can affect patients of any age, gender, and race worldwide [15]. It is characterized by an elevation of immunoglobulin G (IgG) even in the absence of cirrhosis, circulating autoantibodies, association with human leucocyte antigens (HLA) DR3 and DR4, distinctive histological features, and a favorable response to immunosuppression [16,17]. It is a diagnosis of exclusion; thus, viral infections, alcohol consumption, hepatotoxic drugs, or a history of genetic liver disorders must be ruled out prior to establishing the diagnosis [18].

Diverse clinical manifestations of AIH in different patient groups, from asymptomatic disease to acute hepatic failure, pose a diagnostic challenge even for experienced clinicians [15,18]. Heterogenous biochemistry results are an additional difficulty [17].

Some patients with an acute form of AIH may initially present with normal levels of IgG, antinuclear (ANA), and/or negative smooth muscle antibodies (SMAs). A more substantial autoimmune liver serology testing should be considered in those cases, as in some cases, autoantibodies may only turn positive up to a few months after the first symptoms of the disease [17].

Patients can present with an indolent course of the disease; however, acute or asymptomatic onsets are the other expression patterns of the disease [15,18]. The most frequent form is acute onset AIH, where jaundice and significantly elevated transaminase levels are observed [15]. Acute severe hepatitis, which can lead to acute liver failure, affects only a small number of patients, who often require a liver transplant [15].

In the insidious onset form of AIH, either no apparent symptoms or only non-specific manifestations (fatigue, right upper quadrant pain, lethargy, malaise, anorexia, weight loss, nausea, pruritus, fluctuating jaundice, and polyarthralgia involving the small joints without arthritis—sometimes dating years back) are observed [17].

A typical biochemical profile of the disease is usually hepatic. Bilirubin concentration and aminotransferase activity ranging from just above the upper limits of normal to more than 50 times that value are being observed [19,20,21]. Recent studies revealed that gamma-GT levels may also be increased in AIH, which could be used as an independent predictor of treatment outcome [22,23].

AIH in pregnant patients was first described in the 1970s as a nosological entity with unfavorable pregnancy outcomes for both the mother and fetus [24]. Early miscarriage, preterm birth, and low body weight, as well as a large number of CSs, were often reported [24]. The disease is rarely observed during gestation—in pregnant patients with AIH, the improvement or even remission of the disease can be noticed. However, a progression with strong manifestation is sometimes reported in the postpartum period [17]. In the course of AIH, 7–21% of pregnant patients experience flares, which may lead to hepatic decompensation. Maternal complications include pre-eclampsia, disease progression, degeneration of the liver with the need for transplantation, or even death [24]. Nevertheless, more recent studies have undermined the previous findings and reported largely favorable maternal and fetal outcomes [24].

The available treatment options for AIH usually prevent a severe course of the disease, leading to normalization of transaminase and IgG levels within 6 months, defined as remission and being an important prognostic factor for long term well-being [15,18,25]. An excellent prognosis is usually achieved when AIH is treated properly with good response, whereas untreated AIH progresses uncontrollably to liver failure and death within 5 years in most cases [15]. Glucocorticosteroids (GCSs) are the first-line therapy option which improves survival, with azathioprine reportedly maintaining disease remission [18,24,25]. Treatment is often lifelong, and it is the immunosuppression that is responsible for controlling the disease [26]. The management of AIH during gestation is not yet clearly defined; however, GCSs are considered safe during this vulnerable period [7]. Prednisolone should be used and continued during pregnancy in order to prevent flares [18]. However, azathioprine is a category D drug in gestation; thus, its use is not widely accepted [18]. Nonetheless, no adverse effects were reported in pregnant patients with AIH treated with azathioprine; moreover, such effects seem to appear with higher frequency if the drug is not introduced. Both prednisolone and azathioprine can be used safely in the postpartum period [18]. In their case report, Hirokazu et al. treated their AIH pregnant patient with plasma exchange (PE) and continuous hemodiafiltration (CHDF) therapy, as well as steroid pulse therapy and azathioprine, leading to regained liver normalcy [27]. Around 10–20% of AIH patients do not achieve remission or develop dangerous side effects, which requires treatment discontinuation [15]. The only option for worsening patients not responding to pharmacological therapy is liver transplantation [15].

AIH can be associated with other hepatic conditions, in particular cholestatic liver diseases: primary biliary cholangitis (PBC) or primary sclerosing cholangitis (PSC), drug-induced liver injury (DILI), alcoholic and non-alcoholic steatohepatitis (NASH), or viral hepatitis [17]. Some patients with AIH develop “overlap syndrome” in which simultaneously or consecutively to AIH symptoms, clinical, biochemical, serological, and/or histological features of PBC or PSC are observed. To describe these patients, the following terms are used: “the hepatic forms of PBC”, “secondary autoimmune hepatitis”, “autoimmune cholangitis”, “autoimmune sclerosing cholangitis”, or “combined hepatic/cholestatic syndromes” [17,28,29,30,31]. The prevalence of AIH and PBC symptoms simultaneously (AIH-PBC variant) are observed in 8–10% of adult patients with AIH or PBC [17]. The “Paris criteria” are the most common diagnostic tool for the “overlap syndrome” and include the following for PBC: (1) alkaline phosphatase (ALP) ≥ 2× upper normal limit (ULN) or gamma-glutamyl-transpeptidase (gamma-GT) ≥ 5× ULN; (2) presence of antimitochondrial antibodies (AMAs); (3) a liver biopsy specimen showing florid bile duct lesions. For AIH: (1) alanine aminotransferase (ALT) ≥ 5× ULN; (2) serum IgG levels ≥ 2× ULN or the presence of SMAs; (3) a liver biopsy showing moderate or severe periportal or periseptal lymphocytic piecemeal necrosis. At least two of the three accepted described key criteria of each disease must be met to make the AIH-PBC overlap syndrome diagnosis [32], which then has a sensitivity of 92% and specificity of 97% [33].

### 3.2. Primary Biliary Cholangitis (PBC)

Primary biliary cholangitis (PBC) is among the most common causes of cholestatic liver disease [13]. Primary biliary cholangitis (PBC) is a female-predominant, chronic autoimmune liver disease with slow progression over time, which might ultimately lead to cirrhosis and hepatic failure [14,34]. It usually develops in genetically susceptible patients and is triggered by environmental factors [14]. Different bile acid metabolism in females might be a result of distinct sex hormones levels, and it was proven that increased estradiol levels might be partly responsible for cholestasis [34].

PBC is usually diagnosed at peri-menopausal age; nevertheless, as many as 25% of patients might be of childbearing age [34]. In fact, PBC diagnosis in pregnant patients occurs typically in their early 30s [35]. The clinical manifestation might vary slightly between individuals but commonly manifests as pruritus, fatigue, and jaundice [14,35].

Biochemical markers which are an alarm for the early stage of cholestasis are elevated serum alkaline phosphatase (ALP) and gamma-glutamyltranspeptidase (GGT) levels, while in advanced stages, conjugated hyperbilirubinemia might also appear [14]. An increased ALP level is the most important biochemical indicator of PBC in cholestasis; however, it might be caused by placental production during gestation [35]. Additionally, anti-mitochondrial (AMAs) autoantibodies and liver function tests, as well as liver biopsy, are helpful tools in disease detection [35].

In their retrospective analysis, Trivedi et al. reported few patients experiencing pruritus prior to becoming pregnant and before PBC diagnosis, while for over half of patients, pruritus developed during pregnancy as the first clinical symptom [34]. Pruritus is a frequent practical concern in patients with PBC, as well as ICP [34]. While the cause is difficult to recognize, symptom-specific treatment of pruritus is usually required. The therapy usually includes solely antihistamines, cholestyramine monotherapy, cholestyramine together with rifampicin, or all the above. For patients who do not benefit from the pharmacological treatment, ultraviolet light therapy or plasmapheresis might be beneficial [34]. A report by Poupon et al., however, presented proof of successful treatment with ursodeoxycholic acid (UDCA), which resulted in patients’ normal delivery preceded by an uneventful pregnancy period [36].

UDCA is the first-choice therapy for PBC due to its anti-cholestatic and anti-fibrotic effects [14]. The drug is a metabolic by-product of intestinal bacteria, naturally occurs as a tertiary bile acid (BA), and is comprised of 1–3% of the human BA pool [8,12,35]. The toxic bile acids stored in the liver are neutralized by the compound, which inhibits liver cell apoptosis and cholangiocyte proliferation, leading to a significant decrease in biochemical markers, as well as the general improvement of the prognosis [14,35]. UDCA is not officially approved during gestation nor breastfeeding; nevertheless, studies have proven it to be safe in noncirrhotic patients [34].

Efe et al. reported that even though 30% of analyzed pregnant patients with PBC experienced biochemical flares, no maternal deaths occurred [37]. Biochemical flares were not observed in patients who systematically administered UDCA before conception and the large number of women experience normal liver biochemistry throughout pregnancy [34].

It was noticed in previous reports that around 50% of pregnancies with PBC ended in preterm delivery (PTD, before 37 weeks). Even though most of the patients developed a significant biochemical deterioration in the third trimester, no adverse maternal nor fetal events were observed in the article by Trivedi et al. on the maternal and fetal outcomes for pregnant women with PBC [34].

### 3.3. Intrahepatic Cholestasis of Pregnancy (ICP)

Intrahepatic cholestasis of pregnancy (ICP), also known as obstetric cholestasis (OC), is an atypical but most common pregnancy-specific cause of cholestatic liver disease with varied global incidence [12,13]. This reversible form of cholestasis usually arises in the second or third trimester of pregnancy [8,12,13]. The disease has a multifaceted etiology—it might result from an increased estrogen level during gestation or modified expression of hepatobiliary transport proteins [38]. ICP often poses a diagnostic challenge for clinicians, as clinical symptoms tend to precede the deviations in laboratory results, and the clinical features and onset time are similar to PBC characteristics [35,38].

The characteristics of ICP include pruritus, elevated ALP, serum bile acid (SBA), and liver transaminases levels, rarely jaundice, and always a spontaneous resolution of symptoms within a few weeks after delivery [12,38,39]. Apart from the gestation period, patients are usually asymptomatic [8]. Although rash is not a symptom of ICP, patients might present with dermatitis artefacta due to scratching [12]. If the disease does not resolve up to six weeks postpartum, an alternative liver pathology should be considered [12].

The most valued test for the diagnosis is the serum bile acid (SBA) level [12]. A total SBA level of over 40 umol/L showed a correlation with a high incidence of fetal complications [39]. Nevertheless, healthy pregnant patients might present a similar range of total SBA as women with ICP [39]. Most commonly, it is abnormal serum liver tests and increased serum bile acids that point to the diagnosis of ICP in a pregnant patient; however, in a minority of cases, serum bilirubin might be raised [12].

Biochemical abnormalities might justify a decision about earlier delivery [38]. Several guidelines on ICP recommend monitoring disease progression by weekly liver function tests and coagulation tests in the case of abnormal results or before labor [38]. Studies show that maternal BA levels correlate positively with fetal SBA. Raised levels of BA associated with ICP pose a greater risk of spontaneous preterm birth, fetal distress, and stillbirth [38,39]. Although there are many adverse outcomes of pregnancy linked to ICP, the consequences for the mother are usually benign, the biggest complaint being usually the discomfort caused by pruritus [8,12]. Nonetheless, reports point to an increased susceptibility to hepatobiliary pathologies later in life, including chronic hepatitis, cirrhosis, and cholangitis, as well as immune-mediated and cardiovascular diseases [8,12,38].

Clinicians’ opinions regarding the disease’s diagnostic criteria and treatment vary, leading to difficulties in management decisions [38]. The most common and first-line treatment of ICP is ursodeoxycholic acid (UDCA), which was proven to reduce pruritus and decrease abnormalities in laboratory results [8,12,38]. The guidelines on ICP do not agree on the UDCA dosing, which varies from 10–15 mg/kg/day up to 25 mg/kg/day depending on the severity of the disease [38]. In their meta-analysis, Bacq et al. concluded that for the offspring of ICP patients, a decrease in both fetal distress syndrome and neonatal intensive care unit admissions are associated with UDCA administration [40]. However, the effects of in utero exposure to SBA on adverse fetal outcomes need further investigation [12].

### 3.4. Future Perspectives

Acute liver failure (ALF) during pregnancy remains a complex and critical clinical challenge, necessitating further research to enhance diagnostic accuracy, therapeutic strategies, and maternal–fetal outcomes. One promising avenue for future exploration is the role of gut microbiota in the pathophysiology and management of liver diseases. Emerging evidence suggests that fecal microbiota transplantation (FMT) may serve as a potential therapeutic modality in hepatic disorders, specifically cirrhosis [41].

Although current research primarily focuses on non-pregnant populations, there is potential for exploring FMT as an adjunct therapy in managing ALF during pregnancy. Future research should prioritize the evaluation of its safety, efficacy, and optimal administration protocols in pregnant individuals, taking into account the unique physiological adaptations of pregnancy and their implications for maternal and fetal health. Additionally, a deeper understanding of gut microbiota–liver interactions in pregnancy may yield novel insights into the pathogenesis of ALF and identify new therapeutic targets. The integration of such innovative microbiome-based interventions with established diagnostic and treatment modalities has the potential to significantly improve clinical outcomes in this high-risk patient population.

## 4. Conclusions

Acute liver failure in pregnancy remains a diagnostic and therapeutic challenge requiring prompt and thorough investigation. Given the rapid progression and life-threatening nature of liver failure, the early recognition of symptoms, comprehensive diagnostics, and timely multidisciplinary intervention are crucial for improving maternal and fetal outcomes. Future studies should focus on improving diagnostic strategies and treatment protocols for acute liver failure in pregnancy, particularly in cases where no definitive etiology can be established.

## Figures and Tables

**Table 1:** Summary of key literature findings.

Condition	Key Features	Diagnostic Markers	Management Strategies	Pregnancy Outcomes
AIH	Chronic, immune-mediated liver disease causing progressive damage, potential for acute liver failure.	Elevated IgG, ANA, SMA (may be normal in acute form of AIH), Elevated concentration of bilirubin and aminotransferases activity, Diagnosis of exclusion.	First-line therapy: GCS (prednisolone as a first choice), considered safe during pregnancy, Azathioprine—allows to maintain remission, Gamma-GT levels may be used as an independent predictor of treatment outcome.	Increased risk of miscarriage, preterm birth, hepatic decompensation, pre-eclampsia.
PBC	Progressive autoimmune-mediated destruction of intrahepatic bile ducts, leading to cholestasis and fibrosis.	Elevated ALP, GGT, AMA. Elevated ALP—the most important biochemical indicator of PBC—not applicable in pregnancy due to placental production.	UDCA, Pruritus management (antihistamines, cholestyramine, rifampicin).	Potential for biochemical flares, but often stable during pregnancy with UDCA treatment; Approx. 50% of pregnancies complicated by PBC ended in preterm delivery.
ICP	Pregnancy-induced cholestasis with elevated bile acid levels, spontaneous postpartum resolution.	Elevated serum bile acids (SBA) (>40 μmol/L associated with fetal risk), healthy pregnant patients might present similar range of total SBA as women with ICP, Eleveated ALP and liver transaminases.	UDCA, early delivery consideration in severe condition.	Increased risk of preterm birth, fetal distress syndrome, stillbirth.

## Data Availability

The original contributions presented in this study are included in the article; further inquiries can be directed to the corresponding author/s.
